# Diversity and Activity Patterns of Medium‐Sized and Large Terrestrial Mammals in Agroforests of a Peruvian Amazon Rainforest Region

**DOI:** 10.1002/ece3.71997

**Published:** 2025-08-15

**Authors:** Yevgeniya Korol, Paddy Collins, Juan Reynaldo Gallegos, Stacey Hollis, Dirk Hölscher, Manuel Huinga, Christopher Kirkby, Nina Gerber

**Affiliations:** ^1^ Tropical Silviculture and Forest Ecology University of Göttingen Göttingen Germany; ^2^ Fauna Forever Puerto Maldonado Madre de Dios Peru; ^3^ Centre for Biodiversity and Sustainable Land Use University of Göttingen Göttingen Germany; ^4^ Biomas S.A.C Puerto Maldonado Madre de Dios Peru; ^5^ Wildlife Sciences University of Göttingen Göttingen Germany; ^6^ KORA Foundation Ittigen Switzerland

**Keywords:** circadian patterns, land‐sharing conservation, mammal species richness, trapping rate, tropical rainforest, vegetation structure

## Abstract

Agroforests offer potential for biodiversity conservation through a land‐sharing approach. However, it remains uncertain whether they can support medium‐sized and large forest‐dependent terrestrial mammals. We evaluated the diversity and activity patterns of such mammals in agroforests and natural forests in the northern buffer zone of the Tambopata National Reserve in the Peruvian Amazon. For agroforests specifically, we examined the influence of connectivity to the core zone of the reserve, vegetation structure and human presence on mammal diversity and activity. In total, 21 species were recorded using camera traps. Agroforests supported 15 species, significantly fewer than neighbouring forests. Five of the seven threatened species were found exclusively in forests. Nonetheless, one third of the recorded species exhibited similar or higher trapping rates in agroforests, with *Tapirus terrestris* showing rates up to 6.3 times higher than in forests. The diurnality index across cathemeral species was significantly higher in agroforests adjacent to the protected area and marginally so in forests. In agroforests, mammal diversity increased with greater tree DBH, canopy cover and taller understorey vegetation. The trapping rate of *Dasyprocta variegata* also increased with total tree species richness. Agroforests along the Tambopata River can thus support a substantial number of medium‐sized and large terrestrial mammals. However, forests remain critical for conserving mammal species richness, particularly for those of urgent conservation concern. Enhanced vegetation structure in agroforests—particularly larger trees, a denser canopy, and taller understorey vegetation—can increase their value as a habitat for medium‐sized and large forest‐dependent terrestrial mammals.

## Graphical abstract

We studied species richness and activity patterns of medium‐sized and large terrestrial mammals in agroforests located in the northern buffer zone of Tambopata National Reserve, Peru. The agroforests harboured a high number of mammal species, with species richness increasing alongside greater structural complexity of vegetation. However, forests supported significantly more species of medium‐sized and large terrestrial mammals, particularly those of high conservation concern.

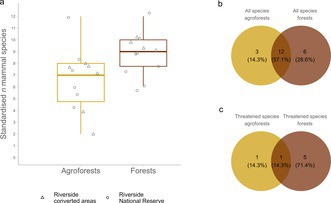

## 1 Introduction

The Amazon rainforest is considered the most biodiverse forest region on Earth (IPBES 2018). However, over the past 35 years, it has undergone the most rapid increase in human impact among all South American biomes, and nearly half of its area is now modified by human activity (Zalles et al. 2021). The conversion of forest to agricultural land and the intensification of farming practices have contributed to an overall decline in regional biodiversity (Decaëns et al. 2018). In both the Amazon and globally, the tension between the need to conserve biodiversity and the demand for productive land to support a growing human population drives the debate between ‘land sparing’ and ‘land sharing’ approaches to conservation (Fischer et al. 2014; Grass et al. 2021; von Wehrden et al. 2014).

In this context, agroforests—where trees coexist spatially with crops and/or livestock (FAO 2015)—offer a promising strategy for land‐sharing conservation, integrating food production and biodiversity protection within the same space and time (Montagnini 2020). Depending on their origin, structure and management (Ollinaho and Kröger 2021), agroforests can reduce pressure on remaining forest fragments, offer habitat for wildlife and enhance landscape connectivity (Bhagwat et al. 2008; McNeely and Schroth 2006; Schroth et al. 2004).

Although not identical in species composition to adjacent forests, agroforestry systems in Brazil and Guatemala have supported relatively high arthropod diversity (Francesconi et al. 2013; Haggar et al. 2019). Similar patterns have been observed for tropical birds, bats (Farneda et al. 2020; Harvey and González Villalobos 2007; Martin et al. 2021) and mammals in Costa Rican banana–cacao agroforests (Harvey et al. 2006). In more traditional and less intensively managed cacao agroforests in Brazil (Cassano et al. 2012) and coffee agroforests in Ethiopia (Etana et al. 2021), mammalian communities were even comparable to those in nearby forests.

Nevertheless, landscape‐scale factors—particularly forest cover and proximity—strongly influence the conservation value of tropical agroforests for mammals (Caudill et al. 2014; Hending et al. 2018), in interaction with the intensity of management practices (Ferreira et al. 2020). Vegetation complexity within production systems has been shown to enhance mammal diversity, as highlighted by Gallina et al. (1996) in Mexican coffee agroforests and de Silva et al. (2020) in Brazilian cacao agroforests. In Mexican coffee agroforests, the presence of medium‐sized and large mammals was closely linked to tree size (Caudill and Rice 2016). Beyond vegetation characteristics at both plot and landscape scales, factors such as human population density and the presence of domestic animals also affect mammal diversity in agroforests (Cassano et al. 2014; Ferreira et al. 2020). For medium‐sized and large mammals in particular, human–wildlife conflict remains a key challenge that can diminish the conservation potential of agroforests (Nyhus and Tilson 2004; Wong et al. 2015).

Biodiversity in the species‐rich Peruvian Tambopata Province is threatened by the expansion of the road network, legal and illegal gold mining, agricultural conversion and population growth (Nicolau et al. 2019; Sánchez‐Cuervo et al. 2020; Swenson et al. 2011). Deforestation also affects the Tambopata National Reserve (Asner and Tupayachi 2017). In the face of growing anthropogenic pressure, diversified agroforests may help mitigate biodiversity loss (Santos, Crouzeilles, and Sansevero 2019). Across the region, we observed numerous agroforests, particularly homegardens and banana–cacao systems containing a mix of other species. Notably, human–wildlife conflicts are often reduced in these agroforests, as many landowners recognise the economic value of ecotourism driven by wildlife sightings (Kirkby et al. 2010; Torres‐Sovero et al. 2012). However, little is known about the conservation value of agroforests in Tambopata Province for medium‐sized and large terrestrial mammals.

In this study, we assessed the conservation potential of agroforests in the northern buffer zone of the Tambopata National Reserve by comparing the diversity and activity patterns of medium‐sized and large terrestrial mammals in agroforests and natural forests. For agroforests specifically, we examined the effects of vegetation structure and human presence on mammal diversity and activity, and also considered agroforest connectivity to the core zone of the reserve. We measured mammalian activity using trapping rate (Rowcliffe et al. 2014) and diurnality index, as variation in daily activity patterns can reflect increased anthropogenic disturbance (Lee et al. 2024; Negret et al. 2023; Ngoprasert et al. 2007).

**FIGURE 1:**
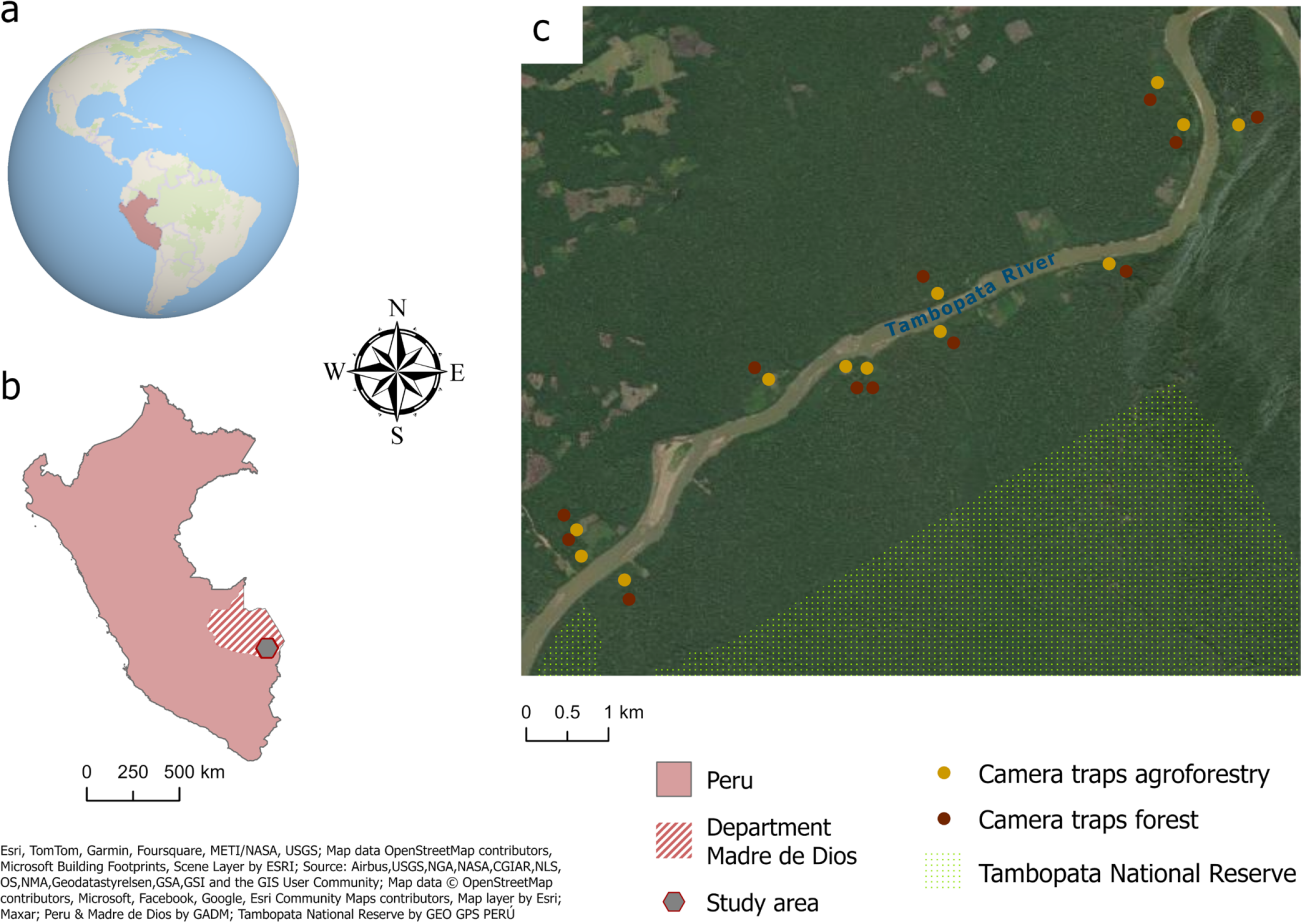
Location of the study area. (a) Peru in the world. (b) Study area location in Peru, Department of Madre de Dios. (c) A zoom into the study area with camera trap locations: orange in agroforests, brown in forests. Tambopata National Reserve in the south. Converted areas in the north and northwest of the image: settlements, slash‐and‐burn agriculture and pastures.

We hypothesised that, despite the relatively high tolerance for wildlife in agroforests within the study area, natural forests would support higher species richness and trapping rates of medium‐sized and large terrestrial mammals. We also expected a higher daylight activity across all species in forests, where individuals are less likely to adjust their activity patterns to avoid human encounters. We further assumed that greater tree diameter, stem density, tree species richness, canopy cover, and understorey height and density would positively influence mammal richness in agroforests, as these vegetation characteristics may increase habitat similarity to natural forests. The Tambopata River crosses the study area, separating the northern terrain from the core zone of the reserve located on the southern bank (Figure 1). Nevertheless, the studied agroforests on the northern side remain embedded within continuous forest, with a mean distance of over 1 km from major disturbances. We therefore expected only a minor influence of riverside location on species richness, trapping rate and diurnality of medium‐sized and large terrestrial mammals in agroforests.

## 2 Methods

### 2.1 Study Area

The study was conducted in the Department of Madre de Dios, Peru, approximately 43 km southwest of the city of Puerto Maldonado, in forests and agroforests along the Tambopata River (12°49′46″ S, 69°26′56″ W–12°53′9″ S, 69°30′24″ W; Figure 1). The region has a mean annual precipitation of 2860 mm and a mean annual temperature of 25.3°C (based on CHELSA climate data; Karger et al. 2017). The dry season extends from April to September. Soils in the area are classified as Plinthic Acrisols (Dijkshoorn et al. 2005). Elevation ranges from 177 to 243 m a.s.l., with a maximum slope of 14°. Continuous forest covers both sides of the Tambopata River in the study region (Figure 1c), adjacent to converted areas on the northern bank and to the core zone of the Tambopata National Reserve on the southern bank. Fieldwork was conducted over a 3‐month period (August–October 2021), coinciding with the early recovery of ecotourism in the area following the COVID‐19 pandemic.

We surveyed six agroforests located on the northern bank of the Tambopata River, adjacent to converted land, and six on the southern bank, adjacent to the Tambopata National Reserve. All agroforests were privately owned, small‐scale (5–10 ha), and varied in age and vegetation structure (Figure S1). Five of the 12 agroforests hosted ecotourism lodges and were otherwise used primarily for subsistence farming. Two were managed for sustainable essential oil production in combination with stingless beekeeping and cultivation of *Vanilla pompona* subsp. *grandiflora* (Lindl.) Soto Arenas. One agroforest belonged to the research station of the NGO Fauna Forever. The remaining four were banana–cacao agroforests. Major cultivated species included arborescent crops such as banana (*Musa* spp.), cacao ( *Theobroma cacao* L.), citrus (*Citrus* spp.), cupuaçu ( *Theobroma grandiflorum* (Willd. ex Spreng.) K. Schum.), *Inga* spp., papaya ( *Carica papaya* L.), peach palm ( *Bactris gasipaes* Kunth) and star fruit ( *Averrhoa carambola* L.).

Each agroforest was paired with a directly adjacent forest site. These forests consisted of a mixture of mature primary and secondary or regenerating forest, marked by the presence of touristic trails and frequent natural disturbances such as windthrow. Tambopata National Reserve was established in 2000. Although the reserve includes a protected core zone, local residents in the surrounding buffer zone are permitted to extract natural resources for subsistence, including bushmeat through hunting.

### 2.2 Camera Traps

We deployed 24 camera traps in a paired design, with each agroforest trap matched to one in an adjacent forest. The maximum distance between paired cameras was 290 m (Figure 1c). The agroforests and forests on the southern bank of the Tambopata River were directly adjacent to the core zone of the Tambopata National Reserve. In contrast, the northern bank was separated from the reserve by the river and more directly connected to converted areas to the north and northwest (Figure 1c). Although the Tambopata River was relatively shallow during the observation time (maximum depth 2 m), it represented a barrier to animal movement. Agroforests on both riverbanks were still embedded in a continuous forest matrix, and the mean distance from northern agroforests to major disturbances exceeded 1 km (Figure 1c). Nevertheless, to account for possible differences, we distributed camera traps equally between the two sides of the river, with an equal number of agroforest–forest camera pairs on each side (hereafter referred to as riverside converted areas and riverside National Reserve).

The minimum distance between two agroforest camera traps was 323 m, with forest patches separating the sites. Agroforest traps were installed on animal paths, as far from buildings as possible, and outside the canopy of neighbouring forests, typically 30 m from the forest edge. To minimise edge effects in forest plots while ensuring regular access for maintenance (every 2 weeks), forest cameras were placed approximately 200 m from the forest edge. On the southern bank, one camera pair was located about 500 m from the core zone of the National Reserve, while the others were situated more than 1 km away.

We randomly assigned six different camera trap models across the study sites (Table S2). Based on prior fieldwork experience, cameras were mounted 50 cm above ground. All cameras used infrared flash and captured three images per trigger, with a 5‐s PIR interval. The shooting angle ranged from 52° to 55° in agroforests and from 40° to 55° in forests. To assess the consistency of performance among camera models, we measured the true detection distance by estimating the furthest position at which the most frequently recorded species (*Dasyprocta variegata*, with 754 trapping events) was detected by each trap. Detection distances ranged from 3.9 to 13.6 m in agroforests and from 3.8 to 9.8 m in forests and did not vary by camera model. Additionally, the correlation between detection distance and mammal species richness was weak (Spearman's *ρ* = 0.01). The correlation with trapping rate was moderate but declined to weak after removing an outlier (Spearman's *ρ* = 0.24).

The survey lasted 73 days, ranging from 44 to 73 days per camera, with a mean of 69 days (Table S2). It covered the transition from the dry to the rainy season (22 August—2 November 2021). Further observations into the rainy season were not possible due to rising river levels. However, an observation period of 30 days is typically sufficient to estimate species richness in tropical regions; seasonality in tropical regions is considered less critical (Kays et al. 2020).

### 2.3 Animal Species

The study area supports a wide range of medium‐sized and large terrestrial mammals, from the abundant brown agouti (*Dasyprocta variegata*) to the elusive giant anteater ( *Myrmecophaga tridactyla* ) and jaguar ( *Panthera onca* ) (Figure S3). To ensure detectability using ground‐based camera traps, we included all terrestrial mammal species equal to or larger in size than the Brazilian rabbit ( *Sylvilagus brasiliensis* ), which has a head‐and‐body length of 230–347 mm (Emmons and Feer 1999). We also included species that primarily forage in the canopy but are known to regularly use ground‐level resources, such as prehensile‐tailed porcupines (*Coendou* spp.) and South American coatis ( *Nasua nasua* ).

Species identification was conducted using ‘Neotropical Rainforest Mammals: A Field Guide’ (Emmons and Feer 1999). Scientific names were validated against the Integrated Taxonomic Information System (ITIS 2024). Species conservation status was verified using the IUCN Red List (IUCN 2024). Prehensile‐tailed porcupines (*Coendou* spp.) and long‐nosed armadillos (*Dasypus* spp.) could only be identified to genus level. However, both *Coendou* species reported from the study area ( *C. bicolor* and *C. prehensilis* ) and both *Dasypus* species ( *D. kappleri* and *D. novemcinctus* ) are classified as Least Concern by the IUCN (2024). Therefore, we assigned the status Least Concern to these genera in our data set.

### 2.4 Collecting Data for Explanatory Variables

**FIGURE 2:**
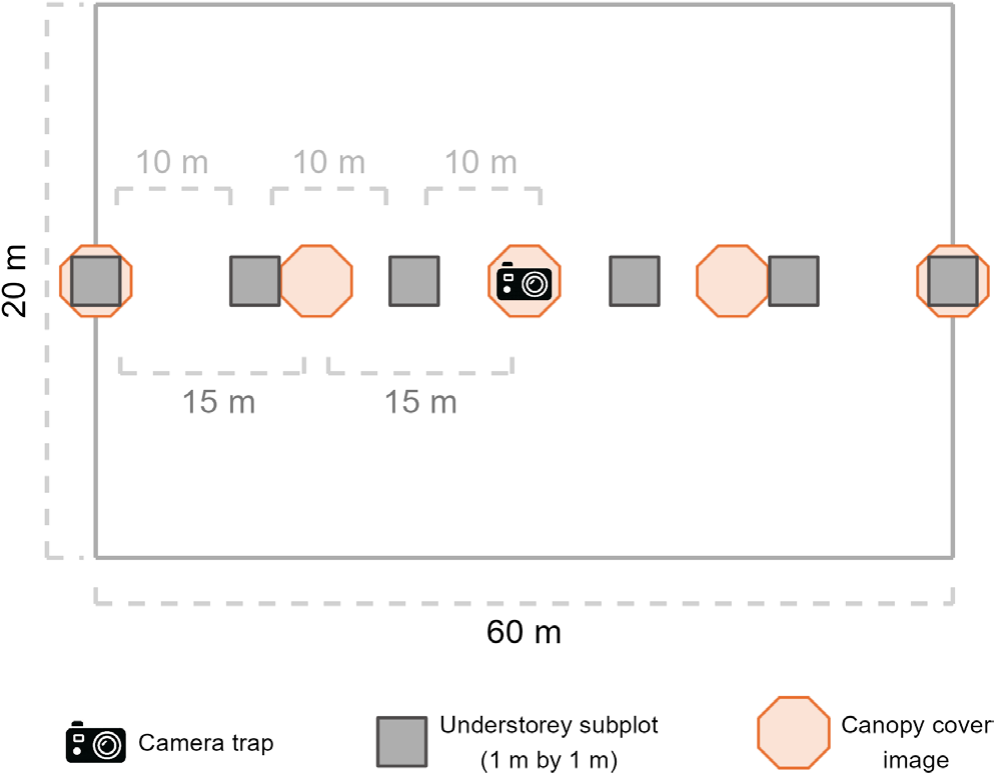
Sampling plot (rectangular 60 m by 20 m) for tree DBH, stem density, canopy cover and plot tree diversity including all arborescent species ≥ 7 cm DBH. Subplots for other measurements — understorey vegetation structure, canopy cover images — were integrated (see figure legend).

We established rectangular plots measuring 60 m in length and 20 m in width, oriented north–south, with each plot centred on the corresponding agroforest camera trap (Figure 2). Within each plot, we recorded the diameter at breast height (DBH) of all arborescent plants with a DBH of ≥ 7 cm, including shrubs and banana plants (*Musa* spp.), and calculated the median tree DBH for each plot. All measured individuals were counted to estimate tree stem density. We identified all arborescent species to compute the Shannon diversity index (Oksanen et al. 2024; Shannon 1948), hereafter referred to as plot tree diversity.

To assess canopy cover, we took square canopy photographs (3000 × 3000 px resolution) using the built‐in camera of a Redmi Note 8T, positioned 2 m above ground level. Photos were taken directly above the camera trap, as well as 15 and 30 m to the north and south of it (Figure 2). Using the R package ‘caiman’ (Díaz 2018), we calculated the mean percentage canopy cover per plot following the method described by Martin et al. (2020).

We assessed understorey foliage density and height—covering grasses, herbs, shrubs and tree saplings under 1.3 m—using a 1 × 1 m portable frame placed in three subplots spaced 10 m apart to the north and three to the south of each agroforest camera trap (Figure 2). We visually estimated foliage density. Understorey height was measured at the centre and corners of each subplot, from which geometric means were calculated per plot.

In addition to plot tree diversity, we considered total tree species richness for each agroforest. This variable extended beyond plot boundaries and more closely reflected the spatial scale relevant to mammal mobility. Data on total tree richness were obtained through interviews with agroforest owners.

The interviews also provided information to assess the intensity of human impact in each agroforest. This included the number of people living in or visiting the site, the number of days per year people were present, the presence or absence of domestic dogs and hunting activity (Table S4). Interviews were conducted in the respondent's native language—Spanish in most cases, and English in two. All participants were informed about the purpose of the data collection and the anonymous nature of data use. Participation was voluntary, with interviewees free to skip questions or terminate the interview at any time. Interviews only began after each participant gave explicit informed consent.

### 2.5 Statistics

#### 2.5.1 Describing Species Richness and Activity

Most diversity metrics rely on data relating to abundance or occupancy (Abrams et al. 2021). However, abundance estimates based on camera trap data are typically subject to bias (e.g., Sollmann et al. 2013), and accurate occupancy estimates often require highly intensive sampling efforts (Kays et al. 2020). Therefore, we measured the diversity of medium‐sized and large terrestrial mammals as species richness. To account for lost observation days due to technical issues at some camera sites (Table S2), we standardised species counts per location by the number of effective observation days at each camera (hereafter, standardised number of mammal species).

Assuming that trapping rate is determined solely by the activity level of a species at a given camera site (Rowcliffe et al. 2014), we quantified activity as the number of independent trapping events per species, standardised by the number of true observation days (standardised number of trapping events). Trapping events of the same species were considered independent if at least 30 min had passed between captures or if a different mammal species appeared in the interval. For diurnality analysis, we assigned a value of zero to trapping events occurring between sunset and sunrise, and a value of one to events between sunrise and sunset, using local sunrise and sunset times from Salidaypuestadelsol (2021). We then calculated the mean diurnality index per plot. This index was first calculated across all recorded species, and then specifically for those known to be nocturnal (Emmons and Feer 1999) but recorded as active during daylight in our dataset. For simplicity, we refer to these species as cathemeral (Table S5).

#### 2.5.2 Effects of Habitat Type (Agroforest vs. Forest) and Riverside

We tested the effects of habitat type (agroforest vs. forest) and riverside (northern bank adjacent to converted areas vs. southern bank adjacent to the Tambopata National Reserve core zone) on species richness and activity of medium‐sized and large terrestrial mammals. For this, we applied linear models with habitat type and riverside as explanatory variables. The response variables were: standardised number of mammal species, standardised number of trapping events, diurnality index for all species and diurnality index for cathemeral species. These models included all camera locations (*n* = 24).

For each species with more than 100 recorded events, we estimated kernel density overlaps of hourly activity to compare daily activity patterns between habitat types (agroforest vs. forest), using the R package *overlap* (Ridout and Linkie 2009). We repeated the procedure to compare activity patterns by riverside. Among the species with more than 100 observations, *Dasyprocta variegata* and *Didelphis marsupialis* had more than 75 observations in each habitat and riverside group. Other species exceeding 100 total observations— *Mazama americana* , *Pecari tajacu* and *Tapirus terrestris* —had fewer than 75 observations in one or more groups. For the first two species, we used estimator Δ4; for the latter three, we applied estimator Δ1 (Meredith and Ridout 2021).

#### 2.5.3 Effects of Agroforestry Characteristics

Using camera locations in agroforests (*n* = 12), we tested the effects of agroforest characteristics on the species richness and activity of medium‐sized and large terrestrial mammals. The explanatory variables included vegetation structure—tree DBH, stem density, percentage canopy cover, plot tree diversity, total tree richness, understorey vegetation height and density—as well as the intensity of human impact.

Intensity of human impact was calculated by multiplying the number of people visiting each agroforest by the number of visit days per year and dividing the result by the shortest distance from the camera trap to nearby buildings. This distance was measured using ArcGIS software (Esri 2024). The resulting index was then weighted according to hunting activity and the presence of domestic dogs in the agroforests. Only two agroforest owners reported hunting. In both cases, hunting was not restricted to the respective agroforests but practiced in wide parts of our study area. Therefore, the effect of hunting in the other agroforests was assumed to be only slightly lower, and the intensity of human impact there was weighted by a factor of 0.9. Since domestic dogs are known to significantly affect wildlife (e.g., Guedes et al. 2021), the index in agroforests without dogs was further weighted by a factor of 0.8. The final formula used was:

$$\begin{array}{c} \left(\frac{\text{number of people}\times \text{number of visit days}}{\text{minimum distance to buildings}}\right)\\ \times 0.9\;\left(\text{if}\;\mathrm{no}\;\text{hunting}\right)\times 0.8\;\left(\text{if}\;\mathrm{no}\;\text{dogs}\right) \end{array}$$

Multicollinearity of the explanatory variables was assessed using correlograms generated with the R package *corrplot* (Wei and Simko 2021). Only variables showing weak‐to‐moderate multicollinearity of *R* < 0.6 were retained (Figure S6). In a further step, we excluded all explanatory variables that showed a weak correlation (*R* ≤ 0.2) with each respective response variable of interest in simple regressions (Figure S7a,b). As fitting models with many predictors and a small sample size (*n* = 12 in this case) can lead to biased results (Burnham and Anderson 2002), we included no more than two explanatory variables in each model (Table S8). Model selection was based on the corrected Akaike Information Criterion (AICc; Akaike 2011), and we considered all models within ΔAICc ≤ 2 of the top‐ranked model (Burnham and Anderson 2002).

For the standardised number of trapping events, we fitted models for all species combined and separately for each species with more than 100 observations. For the diurnality index, models were fitted for all species and separately for cathemeral species. All models were implemented using the lm() function in the *stats* package in R, version 4.3.1 (R Core Team 2023). Results were visualised using the *ggplot2* package (Wickham 2016).

## 3 Results

### 3.1 Species Richness by Habitat Type and Riverside

**FIGURE 3:**
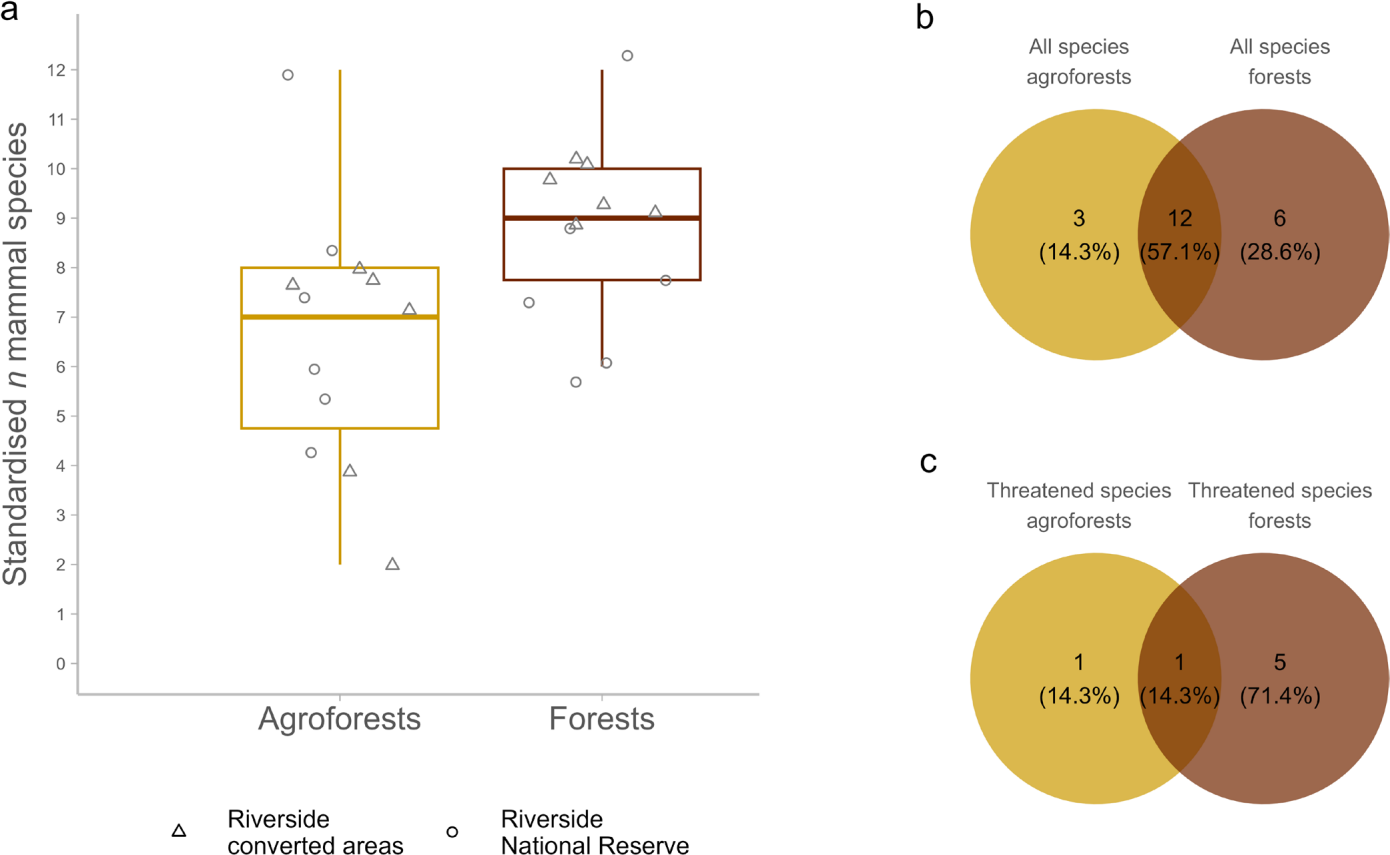
(a) Standardised number of medium‐sized and large terrestrial mammal species by habitat type (agroforests in orange and forests in brown) and riverside. Camera trap locations at the riverside adjacent to converted areas indicated with triangles; camera trap locations at the riverside adjacent to the core zone of Tambopata National Reserve indicated with circles. (b) Venn diagram showing distribution of all recorded species between agroforests and forests. (c) Venn diagram showing distribution of IUCN (2024) listed species between agroforests and forests. In (c), the species shared by both habitat types was *Tapirus terrestris* (VU), the species found only in agroforests was *Sylvilagus brasiliensis* (EN) and the species detected only in forests included *Atelocynus microtis* (NT), *Leopardus wiedii* (NT), *Myrmecophaga tridactyla* (VU), *Panthera onca* (NT) and *Priodontes maximus* (VU).

We recorded 21 species of medium‐sized and large terrestrial mammals, 15 of which were observed in agroforests. The mean standardised number of species across all camera trap locations was 8.16 (±2.48; Table 1). Of the species detected, 12 (57%) were shared between agroforests and forests; three species (14%) occurred only in agroforests, and six species (29%) only in forests (Figure 3b). The species found exclusively in agroforests were *Dactylomys dactylinus* (one trapping event), *Puma concolor* (one trapping event) and *Sylvilagus brasiliensis* (50 trapping events). Species accumulation curves indicate that, although our sampling was not fully exhaustive, it approached a complete inventory of medium‐sized and large terrestrial mammals in the agroforests and forests of the study area (Figure S9).

**TABLE 1:** Means (±standard deviation) for standardised number of medium‐sized and large terrestrial mammal species, standardised number of trapping events, diurnality index of all species and diurnality index of cathemeral species only.

	Agroforests riverside converted areas	Agroforests riverside National Reserve	All agroforests	Forests riverside converted areas	Forests riverside National Reserve	All forests	Total
Std. *n* species	6.24 (±2.44)	7.12 (±2.63)	6.68 (±2.46)	10.06 (±1.09)	9.23 (±1.68)	9.64 (±1.42)	8.16 (±2.48)
Std. *n* events	4.55 (±1.76)	4.99 (±1.88)	4.76 (±1.78)	3.06 (±1.48)	4.01 (±1.61)	3.51 (±1.55)	4.09 (±1.69)
Diurnality all	0.33 (±0.10)	0.40 (±0.12)	0.37 (±0.11)	0.43 (±0.06)	0.47 (±0.09)	0.45 (±0.08)	0.41 (±0.11)
Diurnality cathemeral	0.25 (±0.18)	0.42 (±0.09)	0.33 (±0.18)	0.38 (±0.06)	0.45 (±0.08)	0.42 (±0.08)	0.37 (±0.15)

*Note:* Metrics are provided by habitat type (agroforests vs. forests), including a subdivision by riverside (northern riverside—adjacent to converted areas; southern riverside—adjacent to the core zone of Tambopata National Reserve). Standardised number of mammal species is presented as an arithmetic mean; standardised number of trapping events as a geometric mean; diurnality indices as maximum likelihood estimates of *β*.

A linear model with standardised number of mammal species as a function of habitat type (agroforest vs. forest) and riverside (adjacent to converted areas vs. adjacent to the core zone of the Tambopata National Reserve) showed that forests supported significantly more species than agroforests (Table 2; Figure 3a). Among the recorded species, seven (33%) were categorised as Near Threatened, Vulnerable or Endangered. Of these, 71% were found exclusively in forests (Figure 3c).

**TABLE 2:** Output of the model for the standardised number of medium‐sized and large terrestrial mammals in the study area as a function of habitat type (agroforest vs. forest) and riverside (northern—adjacent to converted areas vs. southern—adjacent to the core zone of the National Reserve).

Explanatory variable	Level	Est.	Std. error	*t* value	*p*
(Intercept)		6.67	0.73	9.18	< 0.01
Habitat	Forest	2.96*	0.84	3.53	< 0.01*
Riverside	Southern	0.02	0.84	0.03	0.98

*Note:* Residual standard error = 2.06 on 21 df; *R* ^2^_mult_. = 0.37, *R* ^2^_adj_. = 0.31; *F* = 6.227, df = 2 and 21; *p* < 0.01. Estimates of explanatory variables significant at *α* < 0.01 in bold with asterisks.

### 3.2 Mammal's Diversity and Agroforest Vegetation Structure

**FIGURE 4:**
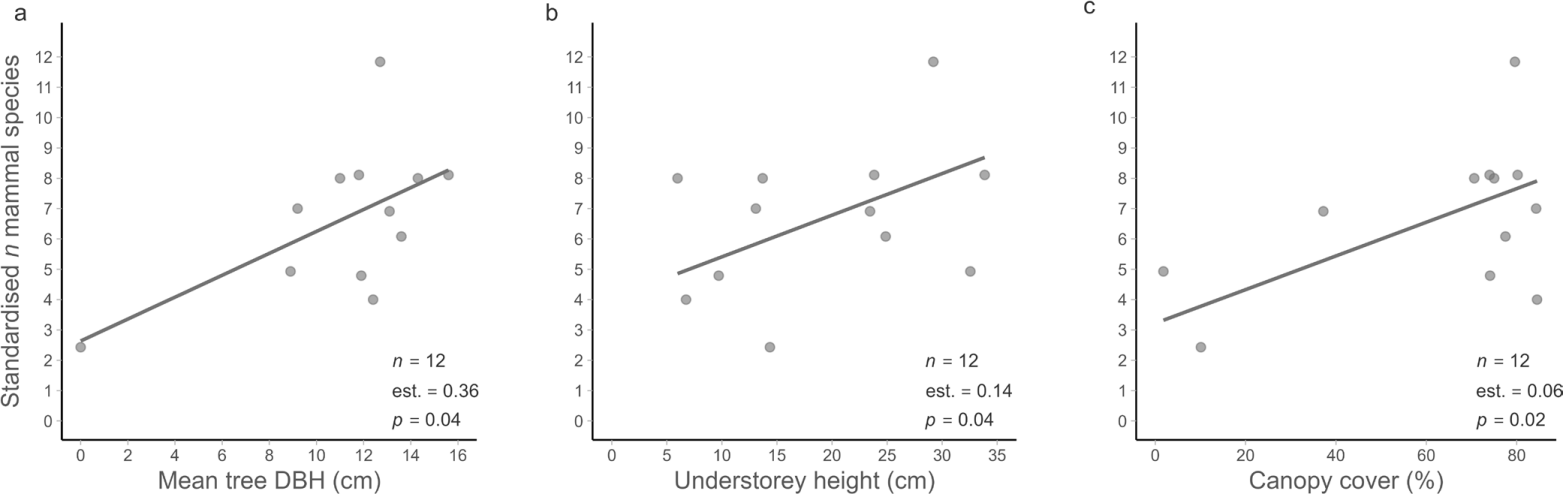
Agroforest vegetation characteristics positively associated with the standardised number of mammal species. (a) Mean tree diameter at breast height; (b) mean understorey height; (c) mean percentage of canopy cover. Coefficients in (a) are based on the best model, with tree DBH as the sole explanatory variable (AICc = 58.49). Coefficients in (b) and (c) are from the second‐best model, which included understorey height and canopy cover as explanatory variables (AICc = 59.16).

The best‐fitting model (AICc = 58.49) indicated that the species richness of medium‐sized and large terrestrial mammals in the studied agroforests increased with tree DBH (Table 3; Figure 4a). The second‐best model (ΔAICc = 0.67) identified understorey height and canopy cover as statistically significant predictors of mammal species richness, with moderate effect sizes. According to this model, an increase of approximately 8 cm in understorey height was associated with the presence of one additional species (Figure 4b). Similarly, species richness increased by 0.06 for every 1% increase in canopy cover, meaning that each additional 16% of canopy cover corresponded to one more mammal species (Table 3; Figure 4c). Also, the fourth competing model indicated a marginally significant effect of canopy cover on the species richness of medium‐sized and large terrestrial mammals (ΔAICc = 1.62; Table 3). The null model ranked third among the competing models (ΔAICc = 1.46; Table 3).

**TABLE 3:** Structure and output of the competing models (ΔAICc ≤ 2) for the standardised number of medium‐sized and large terrestrial mammals in the studied agroforests as a function of agroforest characteristics.

Model structure	Model metrics	AICc	Explanatory variable	Est.	Std. error	*t* value	*p*
~Tree_DBH	RSE = 2.08 on 10 df, *R* ^2^_mult_. = 0.35, *R* ^2^_adj_. = 0.28, *F* = 5.337, df = 1 and 10, *p* = 0.04	58.49	(Intercept)	2.64	1.85	1.42	0.19
			Tree dbh (cm)	**0.36**	0.16	2.31	**0.04**
~Understory height + Canopy cover	RSE = 25.23 on 8 df, *R* ^2^_mult_. = 0.53, *R* ^2^_adj_. = 0.43, *F* = 5.166, df = 2 and 9, *p* = 0.03	59.16	(Intercept)	0.57	1.98	0.29	0.78
			Canopy cover (%)	**0.06**	0.02	2.78	**0.02**
			Understorey height (cm)	**0.14**	0.06	2.33	**0.04**
~1	RSE = 2.46 on 11 df	59.95	(Intercept)	6.68	0.71	9.41	< 0.001
~Canopy cover	RSE = 2.23 on 10 df, *R* ^2^_mult_. = 0.25, *R* ^2^_adj_. = 0.18, *F* = 3.398, df = 1 and 10, *p* = 0.1	60.11	(Intercept)	4.03	1.58	2.56	0.03
			Canopy cover (%)	0.04*	0.02	1.84	0.1*

*Note:* Estimates of explanatory variables significant at *α* = 0.05 are in bold; those significant at *α* = 0.1 are marked with asterisks.

### 3.3 Activity of Mammals: Habitat Type and Riverside

**FIGURE 5:**
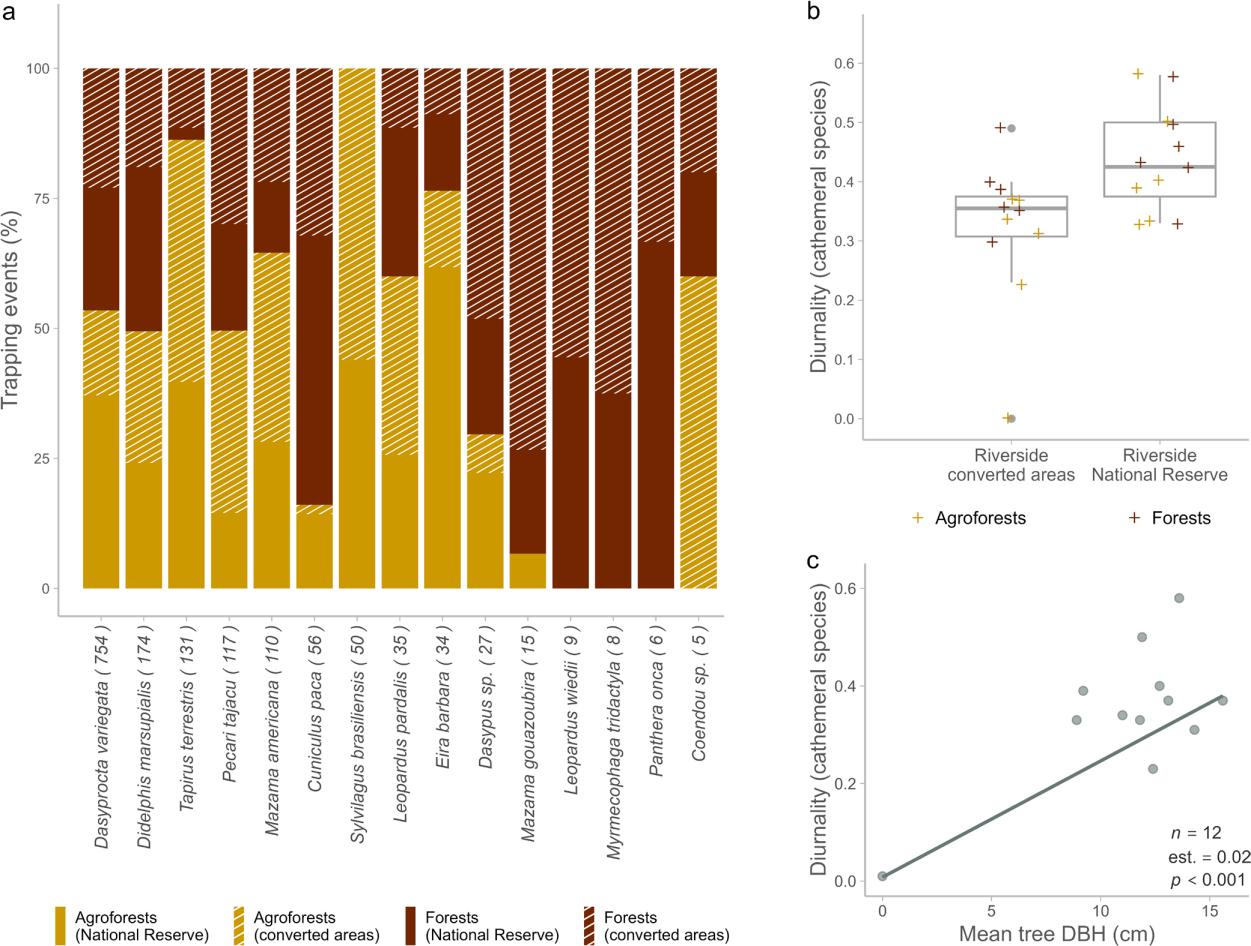
Activity (trapping events). (a) Percentage of trapping events by habitat type (agroforest vs. forest) and riverside (adjacent to converted areas vs. adjacent to the core zone of Tambopata National Reserve) for all species with > 3 independent observations. Numbers in brackets indicate the total number of observations per species. (b) Diurnality index of cathemeral species by habitat type (agroforests in orange and forests in brown) and riverside (adjacent to converted areas vs. adjacent to the core zone of Tambopata National Reserve). (c) Diurnality index of cathemeral mammal species as a function of mean tree DBH. Coefficients in (c) are based on the best model (Table 5).

We recorded a total of 1542 independent trapping events (Table S5), of which 94.7% involved species detected in both agroforests and forests. The mean standardised number of trapping events per camera location was 4.09 (±1.69). The mean diurnality index across all species was 0.41 (±0.11) and 0.37 (±0.15) for cathemeral species (Table 1). Nine of the 15 species with more than three independent observations had approximately half or more of their detections in agroforests. For *Eira barbara* , 76.5% of all observations were in agroforests; for *Tapirus terrestris* , the figure was 86.3% (Figure 5a).

Neither habitat type (agroforest vs. forest) nor riverside (converted vs. reserve‐adjacent) had a significant effect on the standardised number of trapping events across all species and camera locations. We also found no effect of these factors on the overall diurnality index. However, the diurnality index across cathemeral species (Table S5) was significantly higher at the riverside adjacent to the core zone of the National Reserve (*α* = 0.05) and marginally higher in forests (*α* = 0.1) (Table 4; Figure 5b).

**TABLE 4:** Output of the model for the diurnality index of cathemeral medium‐sized and large terrestrial mammals in the study area as a function of habitat type (agroforest vs. forest) and riverside (northern—adjacent to converted areas vs. southern—adjacent to the core zone of the National Reserve).

Explanatory variable	Level	Est.	Std. error	*t* value	*p*
(Intercept)		0.29	0.04	8.24	< 0.01
Habitat	Forest	0.07*	0.04	1.74	< 0.1*
Riverside	Southern	0.11*	0.04	2.72	0.01*

*Note:* Residual standard error = 0.10 on 21 df; *R* ^2^_mult_. = 0.33, *R* ^2^_adj_. = 0.27; *F* = 5.196, df = 2 and 21; *p* = 0.01. Estimates of explanatory variables significant at *α* = 0.01 in bold with asterisks; significant at *α* = 0.1—with asterisks.

**FIGURE 6:**
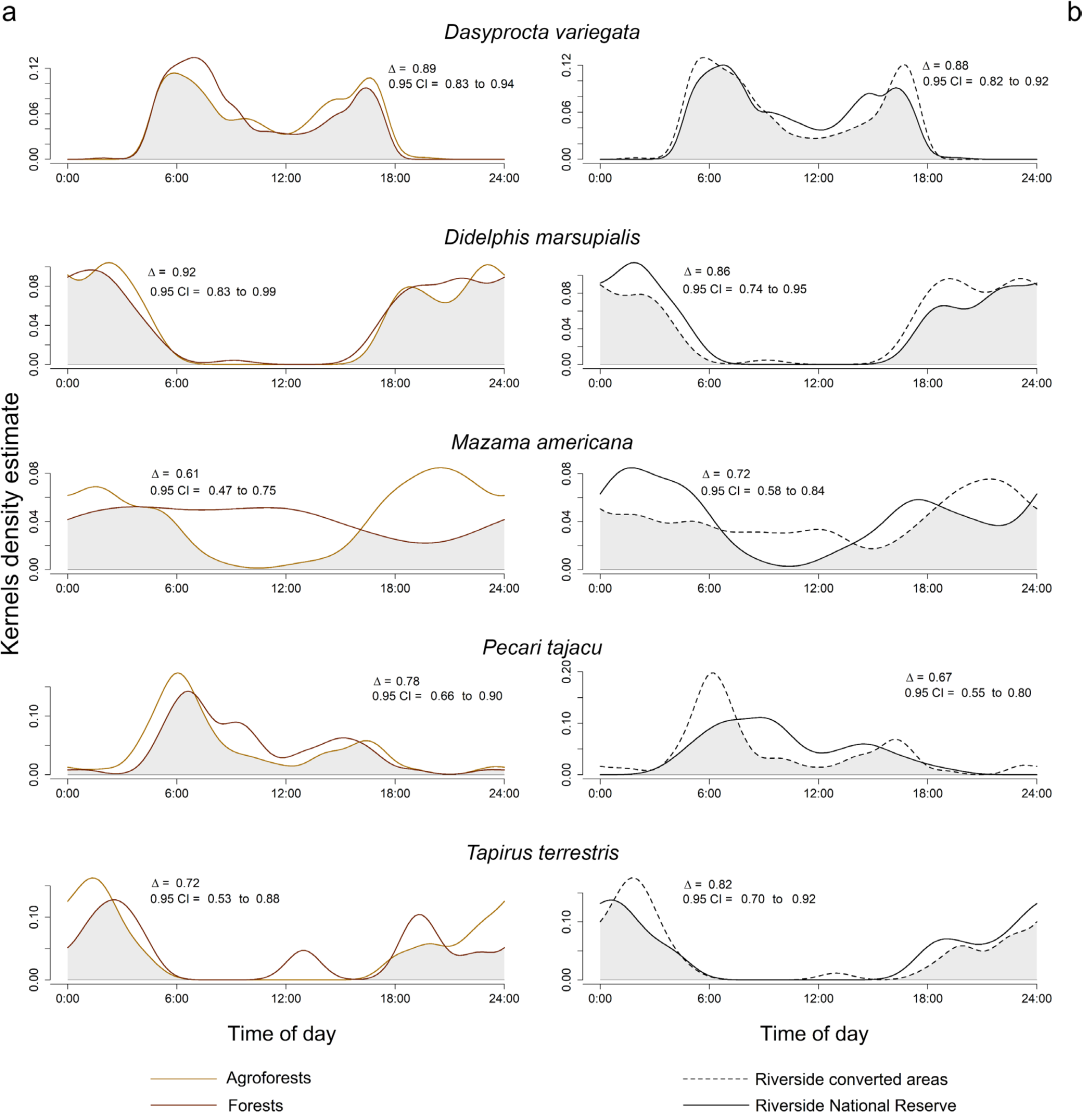
Activity patterns (kernel density estimates) for species having > 100 independent observations. The graphs show overlaps in activity patterns (grey areas beneath the curves) if compared with the activity of the same species by habitat type, that is, agroforest vs. forest in column (a) and if compared to riverside, that is, northern adjacent to converted areas vs. southern adjacent to the core zone of Tambopata National Reserve in column (b).

Kernel density estimates of activity overlap for species with more than 100 independent observations ranged from 0.61 to 0.92 when comparing agroforests and forests (Figure 6a). When comparing riverside types (converted‐ vs. reserve‐adjacent), overlaps ranged from 0.67 to 0.88 (Figure 6b). The greatest difference in daily activity by habitat type was found in *Mazama americana* (overlap = 0.61; 95% CI = 0.47–0.75) and by riverside in *Pecari tajacu* (overlap = 0.67; 95% CI = 0.55–0.80). *M. americana* showed more evenly distributed activity throughout the day in forests than in agroforests, while *P. tajacu* displayed more uniform daily activity at the riverside adjacent to the core zone of the Tambopata National Reserve (Figure 6).

### 3.4 Mammals' Activity, Agroforest Vegetation Structure and Human Impact

We found no effect of agroforest vegetation structure or intensity of human impact on the standardised number of trapping events across all species. However, the trapping rate of *Dasyprocta variegata* in agroforests increased significantly with tree species richness (Table 5; Figure S10). No agroforest characteristics had a significant effect on diurnality across all species. Conversely, the diurnality index of cathemeral species in agroforests increased with tree DBH (Table 5; Figure 5c).

**TABLE 5:** Activity patterns of mammals as a function of agroforest characteristics.

Model structure	Model metrics	Explanatory variable	Est.	Std. error	*t* value	*p*
Events *D. variegata* ~TreeRich_Total	RSE = 27.09 on 9 df, *R* ^2^_mult_. = 0.44, *R* ^2^_adj_. = 0.38, *F* = 7.032, df = 1 and 9, *p* = 0.03, AICc = 111.02	(Intercept)	−3.04	17.19	−0.18	0.86
		Total tree species richness (*n*)	**2.61**	0.98	2.65	**0.03**
Diurn. (cathem. species)~Riverside + Tree_DBH	RSE = 0.07 on 9 df, *R* ^2^_mult_. = 0.79, *R* ^2^_adj_. = 0.74, *F* = 17.020, df = 2 and 9, *p* < 0.01, AICc = 59.16	(Intercept)	0.00	0.07	0.12	0.90
		Riverside Reserve	0.14*	0.04	3.53	< 0.01*
		Tree DBH (cm)	0.02*	0.01	4.52	< 0.01*

*Note:* Here, structure and output of the best model for the standardised number of trapping events of *Dasyprocta variegata* and structure and output of the best model for the diurnality index of cathemeral species. Riverside (northern bank adjacent to converted areas vs. southern bank adjacent to the Tambopata National Reserve core zone) was included in the model for the diurnality index of cathemeral species based on prior analysis, which revealed an effect of riverside on the diurnality of cathemeral species in general (Table 4). Estimates of explanatory variables significant at *α* = 0.05 are in bold; estimates of explanatory variables significant at *α* = 0.001—in bold with asterisks.

## 4 Discussion

We found mammalian communities in agroforests within the buffer zone of the Tambopata National Reserve as relatively species rich, but forests harboured more—in particular threatened—mammal species. Mammal species richness in agroforests increased with tree DBH, canopy cover and understorey height. Animals visited agroforests at a high frequency. Diurnality among cathemeral species increased at the riverside adjacent to the core zone of the Tambopata National Reserve and, to a lesser extent, in forests.

### 4.1 Species Richness in Agroforests

With 15 species recorded, medium‐sized and large terrestrial mammals in the studied agroforests represented a relatively rich group. Caudill and Rice (2016) reported 17 mammalian species in Mexican coffee agroforests, including small mammals. Cassano et al. (2012), however, found more than 20 species of comparable body size in Brazilian *cabrucas*, a native coffee agroecosystem.

Our study was conducted shortly after the end of the COVID‐19 pandemic. Globally, animals were repeatedly reported from areas previously frequented by humans but abandoned during lockdowns (Usui et al. 2021). The relatively high mammal species richness observed in our agroforests may reflect a similar postquarantine effect. It is also possible that wildlife in the study area has developed a degree of tolerance to humans due to the regular presence of tourists, who may be perceived as nonthreatening (Uchida et al. 2024). Further research is needed to evaluate such effects.

We recorded three species—the Amazon bamboo rat ( *Dactylomys dactylinus* ), Brazilian rabbit ( *Sylvilagus brasiliensis* ) and puma ( *Puma concolor* )—exclusively in agroforests. In the case of the Brazilian rabbit, this may be explained by its strong habitat preferences: In forests, it tends to remain near water sources (Emmons and Feer 1999), and only one of our forest cameras was located close to a stream. Similarly, none of the forest cameras faced vegetation of the density or structure preferred by the Amazon bamboo rat (Emmons 1981). As for the puma, this species tends to move along trails in forests (Harmsen et al. 2010) and occurs at generally low densities (Murphy et al. 2022), suggesting that camera traps may need to be placed directly on trails and maintained over longer periods to detect its presence.

Overall, agroforests in our study area supported fewer mammal species than forests, consistent with several synthesis studies showing reduced biodiversity in tropical agroforests compared with forest habitats (Santos, Crouzeilles, and Sansevero 2019; Scales and Marsden 2008). Nonetheless, shade‐rich and less intensively managed cacao and coffee agroforests in tropical regions have sometimes been found to support mammal diversity comparable to that of forests (Cassano et al. 2012; Caudill et al. 2015; Etana et al. 2021; Ferreira et al. 2020). Interestingly, Harvey et al. (2006) found that plantain agroforests in two Costa Rican indigenous reserves supported mammal communities more similar to those in forests than did cacao agroforests. In our study area, plantain and banana (*Musa* spp.) were common. Nevertheless, despite clear foraging opportunities, banana‐dominated agroforests in our study area may offer a vegetation structure less suitable for mammals.

For example, in Mexican coffee agroforests, the presence of large trees was positively correlated with the richness of medium‐sized and large mammals—even when arboreal mammal species were excluded from the analysis (Caudill and Rice 2016). In our study, tree DBH also enhanced mammal species richness. Additionally, species richness increased significantly with both canopy cover and understorey height (Table 3; Figure 4). Denser canopy cover directly supports arboreal mammals (Cassano et al. 2014) and was found beneficial for small nonvolant mammals (Caudill et al. 2015). The positive effect of denser canopy on medium‐sized and large terrestrial mammals in our study might be explained by associated microclimatic conditions (Slater et al. 2024). Taller understorey vegetation provides more shelter but may also be linked to specific plants such as *Heliconia* spp. known to attract species like *Mazama americana* (Emmons and Feer 1999).

Our best models (ΔAICc ≤ 2), fitted to test the effects of agroforest characteristics on mammal species richness, included the null model, and the sample size was relatively small. We therefore cannot draw firm conclusions from these findings. Also, differences in the surrounding landscape matrix can influence how mammals use agroforests (Caudill et al. 2014; Ferreira et al. 2020; Hending et al. 2018). However, our camera traps were placed in a relatively homogeneous landscape at moderate distances from each other. It is therefore likely that variation in mammal richness among the studied agroforests was driven primarily by internal vegetation characteristics. Our findings suggest that structurally complex vegetation enhances the conservation value of agroforests, aligning with conclusions from other studies (e.g., Yashmita‐Ulman et al. 2021).

### 4.2 Threatened Species

Six of the 18 species recorded in forests were categorised as Near Threatened or Vulnerable (IUCN 2024; Figure 3c). In contrast, only two of the 15 species detected in agroforests were of high conservation concern: the Brazilian rabbit ( *Sylvilagus brasiliensis* ) and the Brazilian tapir ( *Tapirus terrestris* ). We observed *S. brasiliensis* , which is categorised as Endangered (IUCN 2024), exclusively in agroforests. This finding appears to contradict a study from southern Brazil, which reported *S. brasiliensis* to avoid human‐dominated areas (Pasqualotto et al. 2024). However, the species is also known to prefer grass vegetation in cultivated landscapes and to be attracted by salt in human urine (Emmons and Feer 1999). Additionally, we observed the Brazilian rabbit to be relatively abundant in the study area. As discussed above, its absence from our forest sample is likely attributable to our camera placement, which may not have aligned with the species' strong preferences for habitats with available streams. The Brazilian tapir ( *T. terrestris* ), a known frugivore (Bodmer 1990; Tobler et al. 2010), was likely drawn to agroforests by the availability of fruit‐bearing plants.

More broadly, most species found in agroforests were also reported from more intensively managed landscapes. Naughton‐Treves et al. (2003) recorded 10 of the 15 species we observed in slash‐and‐burn agricultural fields in the Tambopata Province, northwest of our study area. Similarly, Daily et al. (2003) listed eight of these species in agricultural landscapes in southern Costa Rica.

### 4.3 Mammals' Activity Patterns

We did not find any significant effect of habitat type (agroforest vs. forest) on the standardised number of trapping events or the diurnality index across all species. However, the diurnality index of cathemeral species was significantly higher on the southern riverside, adjacent to the core zone of the Tambopata National Reserve, and marginally higher in forests (Figure 5b). This suggests that mammals on the riverside adjacent to converted areas may adjust their daily activity cycles to reduce the likelihood of encountering humans (Lee et al. 2024; Negret et al. 2023; Ngoprasert et al. 2007). In this context, it is surprising that the intensity of human impact across the studied agroforests had no measurable effect on the circadian rhythms of the focal mammal group. However, Liu et al. (2025) recently reported that mammal communities tended to become more nocturnal in response to ecological changes in human‐modified landscapes, rather than to direct human presence. It is worth noting that the mean distance between camera traps and major disturbances in our study exceeded 1 km. This suggests that the effects of landscape conversion on mammalian circadian rhythms can operate across large spatial scales. In this context, for example, degradation of buffer zones around protected areas (de Almeida‐Rocha and Peres 2021) could potentially alter activity patterns of mammal communities even within the core zones of reserves. Regarding agroforest vegetation structure, the diurnality index of cathemeral species in our study increased with the DBH of agroforest trees. However, the available data are insufficient to support further conclusions on this topic.

Many species in our study were predominantly active in agroforests (Figure 5a). In particular, the high activity of *Eira barbara*, *Tapirus terrestris* and *Sylvilagus brasiliensis* may be linked to their omnivorous, frugivorous and grazing diets, which are likely favoured by the foraging opportunities in agroforests. Although our agroforest plots were located closer to the river than forest plots (205 vs. 425 m on average), and species such as *T. terrestris* may be attracted to water, we consider water proximity to be a minor factor. First, the shortest distance between an agroforest camera trap and the Tambopata River was 97 m. Second, mammals clearly used agroforests for foraging (Figure S3a,b), and tree crop diversity strongly positively explained the number of trapping events, for example, for the brown agouti (Table 5; Figure S10). Fruiting trees have previously been suggested as a means of enhancing the conservation value of agroforests (Gallina et al. 1996). They can increase mammal diversity and abundance, even when planted directly within natural forests, as shown in central Malaysia (Moore et al. 2016).

Among predators, only the ocelot ( *Leopardus pardalis* ) was active in agroforests. *Puma concolor* was recorded once in an agroforest, while other predators—including *Leopardus wiedii* , *Panthera onca* and *Puma yagouaroundi* —were detected exclusively in forests. These species are known to alter their spatial and temporal behaviour to avoid humans (Figel et al. 2021), although the jaguarundi can tolerate smaller‐scale human disturbances (Coronado‐Quibrera et al. 2019). A lower abundance of predators may contribute to increased activity of herbivorous and frugivorous species in agroforests, as prey animals modify their behaviour based on trade‐offs between nutritional gain of an area and predation risk (Griffiths et al. 2025). As for encounters with humans and domestic dogs when foraging in agroforests, wild animals may reduce this risk by adjusting their temporal activity patterns (Gálvez et al. 2024; Manzo et al. 2025). For example, cathemeral species in our studied agroforests were slightly less active by daylight than in forests. The absence of larger felids may also explain the high activity of ocelots in agroforests, although a study across eight Neotropical forests found no negative correlation between ocelot occupancy and the presence of larger predators (Santos, Carbone, et al. 2019).

## 5 Conclusions

Agroforests in the northern buffer zone of the Tambopata National Reserve supported a considerable diversity of medium‐sized and large terrestrial mammals. Nearly half of all detected species—primarily grazing and frugivorous—were observed more frequently in agroforests than in forests. Species richness of mammals in agroforests was higher where vegetation structure was more complex, particularly with increased tree DBH, tree canopy cover and understorey height.

Forests, however, harboured significantly more species overall, and threatened species were found almost exclusively in forested areas. Thus, forest preservation and protection remain the primary conservation strategy to prevent the erosion and extinction of species‐rich mammal communities in Tambopata Province.

Agroforests—especially those with diverse tree crops—can serve as a complementary tool for the conservation of medium‐sized and large terrestrial mammals. The effectiveness of this tool may be enhanced by managing agroforest vegetation to promote greater structural complexity in both canopy cover and understorey.

## Author Contributions

**Yevgeniya Korol:** formal analysis (lead), funding acquisition (lead), visualization (lead), writing – original draft (lead). **Paddy Collins:** investigation (equal), methodology (supporting), writing – review and editing (supporting). **Juan Reynaldo Gallegos:** investigation (equal), writing – review and editing (supporting). **Stacey Hollis:** investigation (equal), writing – review and editing (equal). **Dirk Hölscher:** methodology (equal), writing – review and editing (equal). **Manuel Huinga:** investigation (equal), writing – review and editing (supporting). **Christopher Kirkby:** conceptualization (equal), project administration (lead), writing – review and editing (supporting). **Nina Gerber:** conceptualization (lead), funding acquisition (equal), methodology (lead), writing – original draft (equal), writing – review and editing (lead).

## Conflicts of Interest

The authors declare no conflicts of interest.

## Supporting information


**Data S1:** ece371997‐sup‐0001‐Supinfo.pdf.

## Data Availability

The data that support the findings of this study are openly available at Open Science Framework at https://doi.org/10.17605/osf.io/4x2hm.
