# Supplementary material for: Diversity and Activity Patterns of Medium‐Sized and Large Terrestrial Mammals in Agroforests of a Peruvian Amazon Rainforest Region
Source: Ecol Evol. 2025 Aug 15;15(8):e71997. doi: 10.1002/ece3.71997 (PMC12355011; doi:10.1002/ece3.71997)
Supplement: Supplementary file 1 — Data S1: ece371997‐sup‐0001‐Supinfo.pdf. [file ECE3-15-e71997-s001.pdf]

Supplementary information

Figure S1. 1
Table S2. 2
Figure S3. 3
Table S4. 4
Table S5. 5
Figure S6. 6
Figure S7a. 7
Figure S7b. 8
Table S8. 9
Figure S9. 10
Figure S10. 11

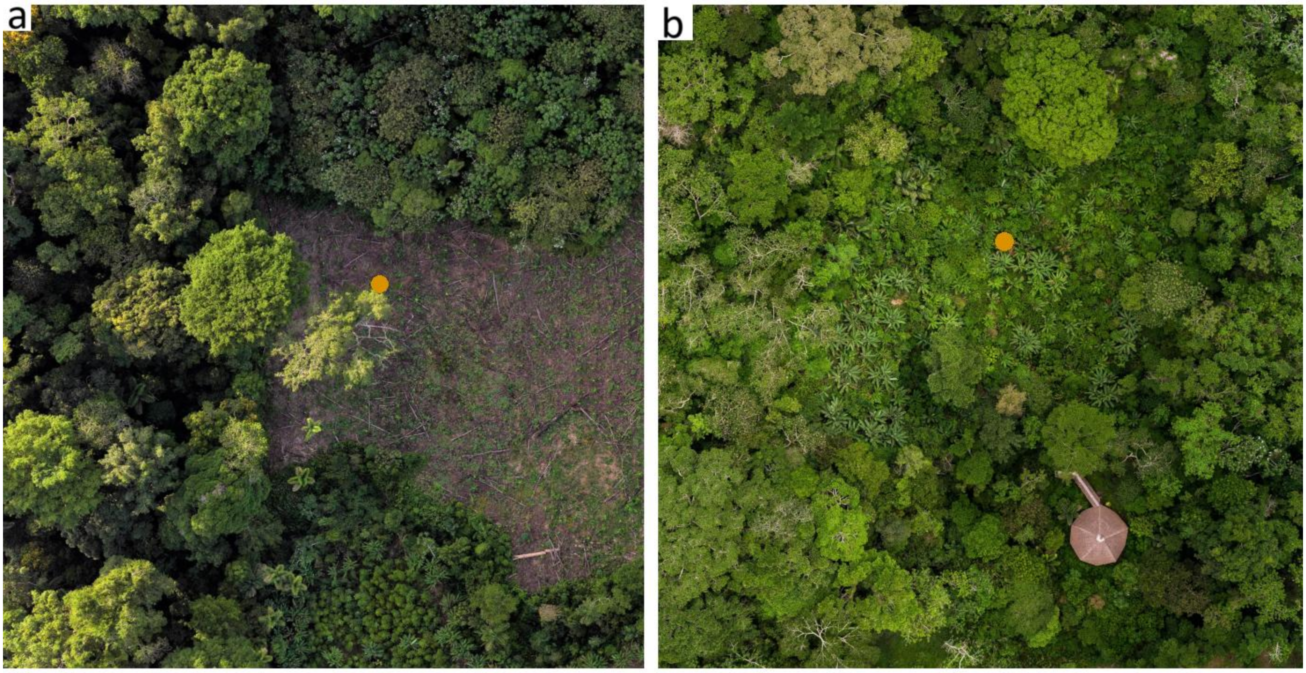

Figure S1. An aerial view of two of the twelve agroforests studied (© Henry Espino Contreras). Orange dots indicate the locations of camera traps in these two estates. (a) One camera is installed in a recently established agroforest with young banana plants. (b) Another camera is installed beneath mature banana plants within a matrix of fruit trees near a building used for tourism.

Table S2. List of camera traps used in the study. The table includes the following columns: Location = ID of the camera trap; Location_Type = code indicating whether the camera was placed in an agroforest or forest; Side = specifies whether the camera was located on the northern bank of the Tambopata River, adjacent to disturbed areas, or on the southern bank, adjacent to the core zone of Tambopata National Reserve; Camera = camera trap model; Obs_Days = number of observation days, excluding days when camera function was impaired; Max_Distance_[m] = maximum detection distance in metres; Shooting_Angle_[°] = camera shooting angle.

**Location**	**Location_Type**	**Side**	**Camera**	**Obs_Days**	**Max_Distance_[m]**	**Shooting_Angle_[°]**
Agr_01	Agroforest	reserve	Browning BTC-5HDPX	72	6.1	55
Fo_01	Forest	reserve	Vision UV 557	72	3.8	52
Agr_02	Agroforest	disturbed	Apeman H55	60	5	55
Fo_02	Forest	disturbed	Vision UV 557	73	4.1	52
Agr_03	Agroforest	disturbed	Browning BTC-5HDPX	73	13.6	55
Fo_03	Forest	disturbed	Apeman H55	73	9.5	55
Agr_04	Agroforest	disturbed	Apeman H55	73	6.6	55
Fo_04	Forest	disturbed	Victure HC 300	64	6.6	40
Agr_05	Agroforest	reserve	Apeman H55	74	6	55
Fo_05	Forest	reserve	Victure HC 300	74	4.5	55
Agr_06	Agroforest	reserve	Browning BTC-5HDPX	73	3.9	55
Fo_06	Forest	reserve	Apeman H55	44	4.9	55
Agr_07	Agroforest	reserve	Victure HC 300	61	11.4	52
Fo_07	Forest	reserve	Apeman H55	73	9.8	55
Agr_08	Agroforest	disturbed	Apeman H55	73	5.7	55
Fo_08	Forest	disturbed	Apeman H55	73	7.6	55
Agr_09	Agroforest	reserve	Victure HC 300	72	6.8	55
Fo_09	Forest	reserve	Browning BTC-5HDPX	72	5.3	55
Agr_10	Agroforest	reserve	Victure HC 300	74	5.2	55
Fo_10	Forest	reserve	Browning BTC-8E	47	6.2	55
Agr_11	Agroforest	disturbed	Apeman H55	74	8.1	55
Fo_11	Forest	disturbed	Browning BTC-5HDPX	61	6.1	55
Agr_12	Agroforest	disturbed	Vision UV 557	72	5.7	52
Fo_12	Forest	disturbed	Victure HC 300	72	5.1	55

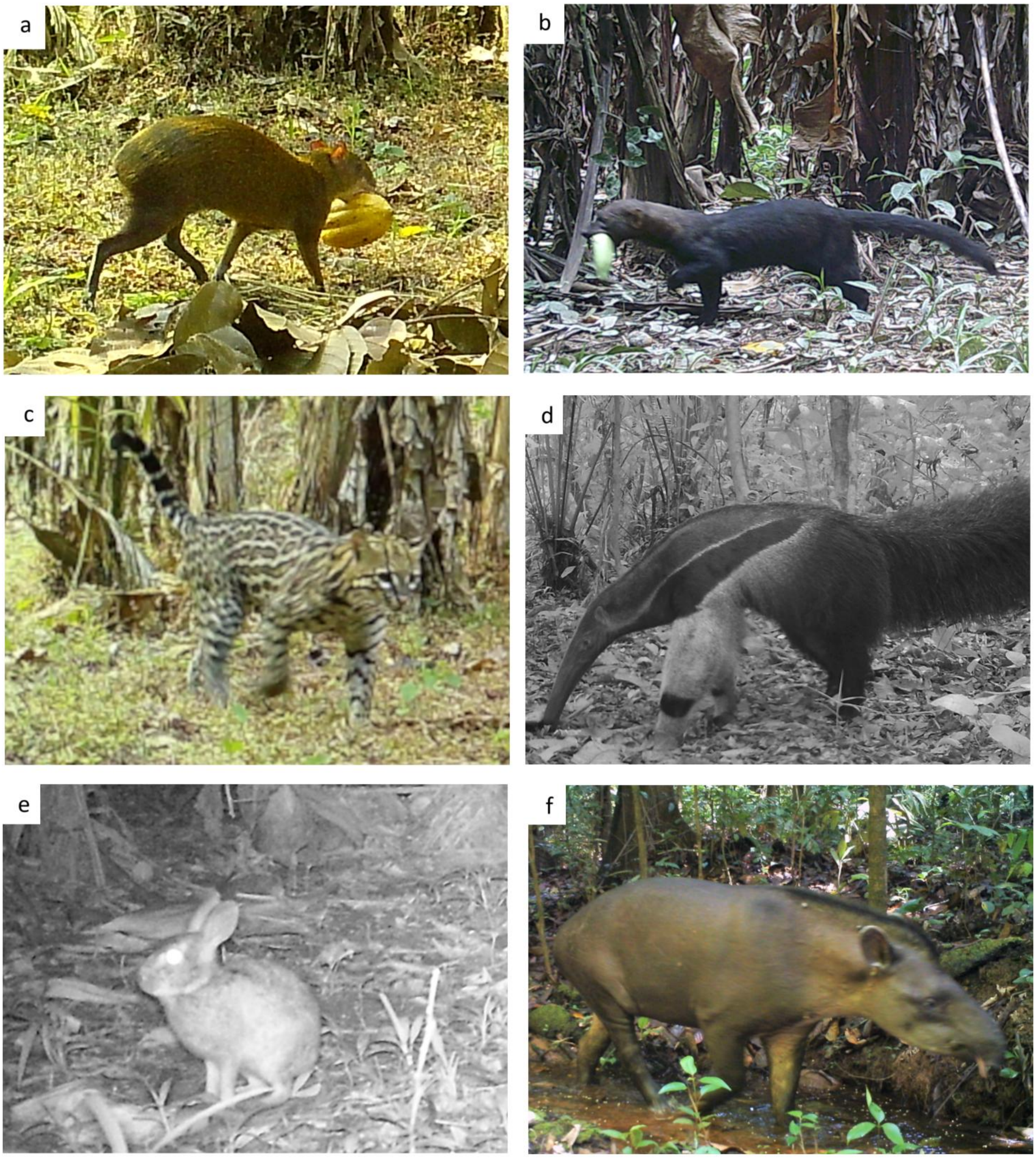

Figure S3. Selected species included in the study: (a) *Dasyprocta variegata* (brown agouti); (b) *Eira barbara* (tayra); (c) *Leopardus pardalis* (ocelot); (d) *Myrmecophaga tridactyla* (giant anteater); (e) *Sylvilagus brasiliensis* (Brazilian rabbit or tapeti); (f) *Tapirus terrestris* (Brazilian tapir). Images (a), (b), (c) and (e) were taken with camera traps in the agroforests studied; images (d) and (f) in forests studied.

Table S4. Questions for interviews with the agroforest owners (translated in English):

1. When did you establish your homegarden?

2. What was it before?

3. Was fire used to establish your farm?

4. Do you use fire in your farm nowadays for silvicultural purposes? If yes, how often?

5. Do you or your workers permanently live at the farm?

6. How many people are there usually?

7. If you do not live at the farm, how often do you or your workers visit it?

8. What herbaceous crops are you growing now?

9. What tree species do you have in your homegarden? How many individuals?

10. Do you use the trees in your agroforest only for edible fruits? Or are the trees useful also for other purposes?

11. Do you apply chemical fertilizers in your homegarden? How much per hectare? How often?

12. Do you apply chemical pesticides? How much per hectare? How often?

13. Do wild animals sometimes cause damages?

To crops?

To trees?

To domestic animals?

14. Are there sometimes hunting events here in the agroforest or near there?

15. How often are there hunting events?

16. Do you or your workers eat some wild animals? If yes, which ones?

17. Do you or your workers eat some wild birds? If yes, which ones?

18. Do you have dogs? How many?

19. Do you tolerate snakes in your farm?

20. If you kill snakes in your farm, then all or selectively only certain species?

Table S5. List of medium-sized and large terrestrial mammals observed during the study. The column *Diurnality* indicates, whether the species was observed during the day only (diurnal), during the night only (nocturnal) or both during the day and during the night (cathemeral).

No.	Species	Common name	Diurnality	Observations *n* (= *n* of independent trapping events)
1	*Atelocynus microtis*	short-eared dog	diurnal	3
2	*Dactylomys dactylinus*	Amazon bamboo rat	diurnal	1
3	*Eira barbara*	tayra	diurnal	34
4	*Mazama gouazoubira*	grey brocket	diurnal	15
5	*Myrmecophaga tridactyla*	giant anteater	diurnal	8
6	*Nasua nasua*	South American coati	diurnal	3
7	*Puma yagouaroundi*	jaguarundi	diurnal	2
8	*Coendou* sp.	prehensile-tailed porcupine	nocturnal	5
9	*Cuniculus paca*	spotted paca	nocturnal	56
10	*Dasypus* sp.	long-nosed armadillo	nocturnal	27
11	*Priodontes_maximus*	giant armadillo	nocturnal	1
12	*Puma concolor*	puma	nocturnal	1
13	*Sylvilagus brasiliensis*	Brazilian rabbit	nocturnal	50
14	*Dasyprocta variegata*	brown agouti	cathemeral	754
15	*Didelphis marsupialis*	common opossum	cathemeral	174
16	*Leopardus pardalis*	ocelot	cathemeral	35
17	*Leopardus wiedii*	margay	cathemeral	9
18	*Mazama americana*	red brocket	cathemeral	110
19	*Panthera onca*	jaguar	cathemeral	6
20	*Pecari tajacu*	collared peccary	cathemeral	117
21	*Tapirus terrestris*	Brazilian tapir	cathemeral	131

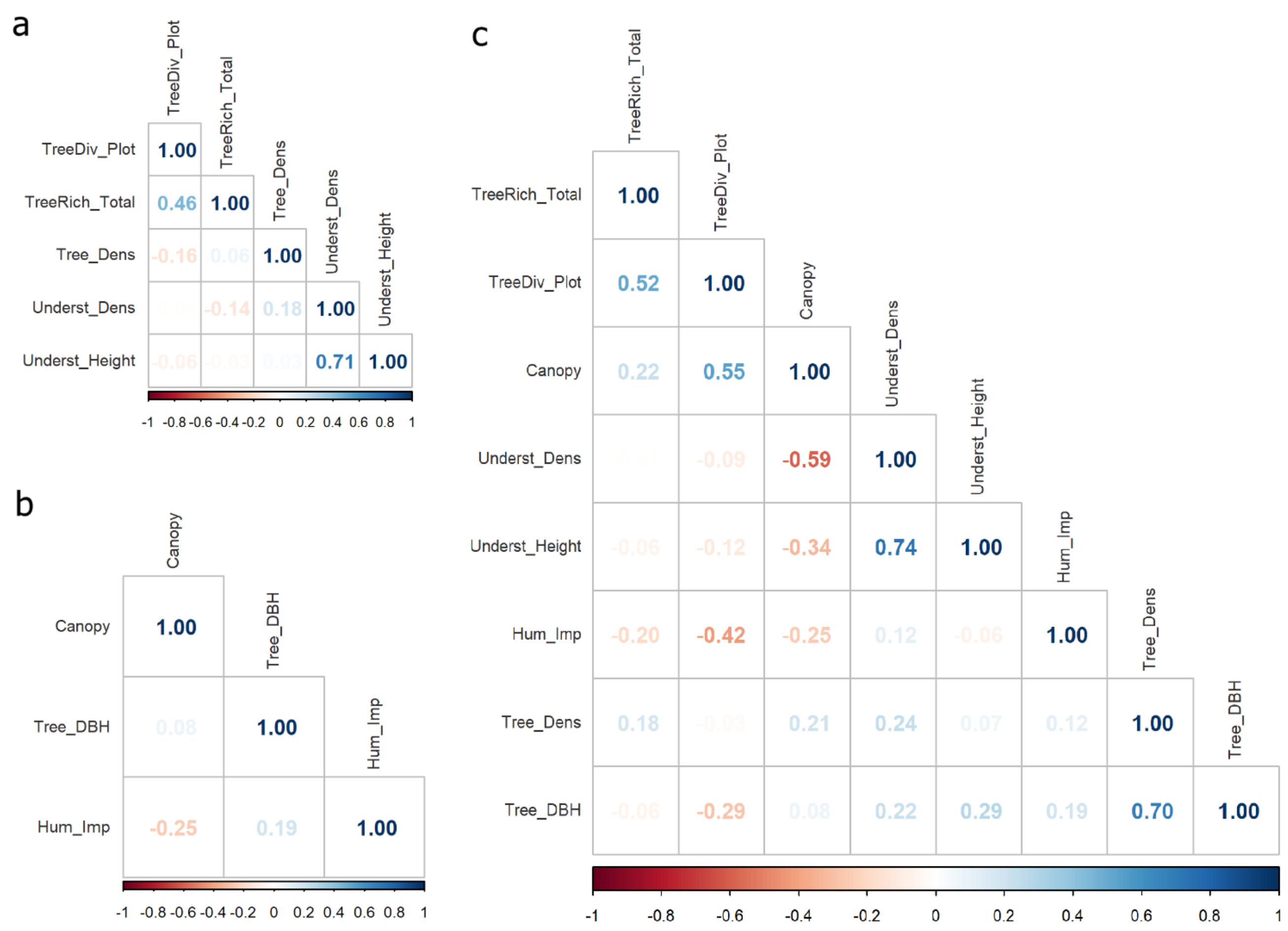

Figure S6. Correlograms of agroforest characteristics. (a) Variables with normal distribution tested with Pearson' correlation: *TreeDiv_Plot* – tree diversity within plots around the camera traps (Shannon), *TreeRich_Total* – Tree richness in the whole agroforest (*n*), *Tree_Dens* – density of arborescent vegetation (stems/ha), *Underst_Dens* – density of understorey vegetation (%), *Underst_Height* – height of understorey vegetation (cm). (b) Variables with non-normal distribution tested with Spearman's correlation: *Canopy* – canopy cover (%), *Tree_DBH* – mean diameter at breast height of arborescent vegetation (cm), *Hum_Imp* – Intensity of human impact (index). (c) Correlogram of all variables to visualise multicollinearity of normally distributed variables with non-normally distributed variables, tested with Spearman's correlation.

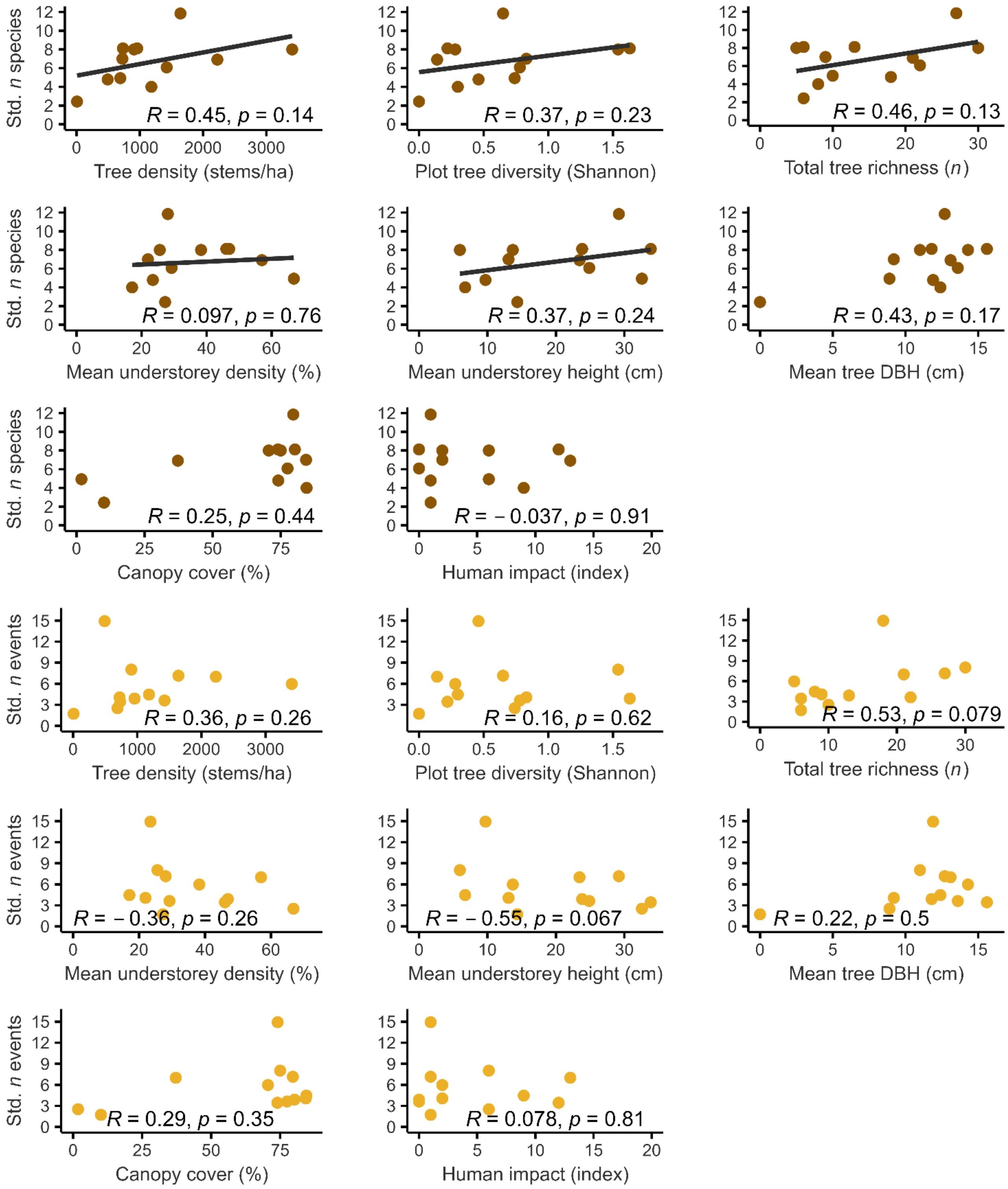

Figure S7a. Simple regressions to test and exclude the weakest (R ≤ 0.2) explanatory variables prior to modelling variables of interest as a function of agroforest characteristics. Variables of interest: standardised *n* of mammal species (in brown) and standardised *n* of trapping events (in yellow). Standardised *n* of trapping events was non-normally distributed, so that respective correlations were tested with the method “spearman”. Among the explanatory variables, mean tree DBH, canopy cover and human impact were non-normally distributed and thus always tested with the method “spearman”.

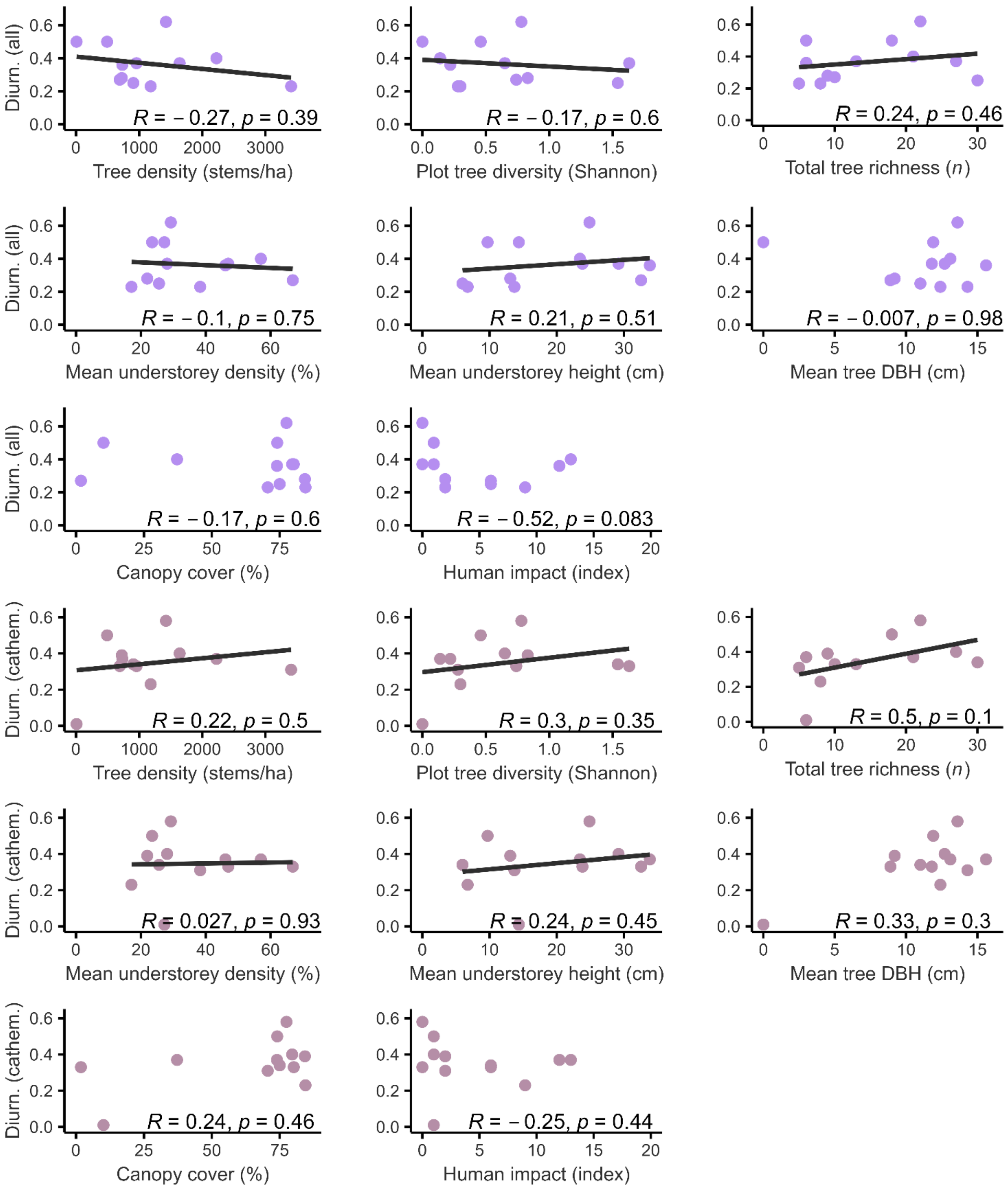

Figure S7b. Simple regressions to test and exclude the weakest (R ≤ 0.2) explanatory variables prior to modelling variables of interest as a function of agroforest characteristics (continued). Variables of interest: diurnality index of all observed species (in bright violet); diurnality index of cathemeral species only (in dust lavender). Among the explanatory variables, mean tree DBH, canopy cover and human impact were non-normally distributed and thus always tested with the method "spearman".

Table S8. Agroforest characteristics considered as explanatory variables for standardised number of mammal species, standardised number of trapping events, diurnality index of all species and diurnality index of cathemeral species. Explanatory variables that had a weak simple correlation with the respective variable of interest ($R \leq 0.2$) were excluded in a preliminary step (Appendix 7a-b). Due to a rather small sample size ($n = 12$), no more than two explanatory variables were included in each model. The explanatory variables that showed a high multicollinearity ($R \geq 0.6$; Appendix 6) were not included in the same model.

Variable of interest	Explanatory variables
Std. *n* species	- Tree density<br>- Tree DBH<br>- Plot tree diversity<br>- Total tree richness<br>- Understorey height<br>- Canopy cover
Std. *n* events	- Total tree richness<br>- Tree density<br>- Canopy cover<br>- Understorey height<br>- Understorey density
Diurnality (all)	- Tree density<br>- Total tree richness<br>- Understorey height<br>- Intensity of human impact
Diurnality (cathemeral)	- Tree density<br>- Tree DBH<br>- Plot tree diversity<br>- Total tree richness<br>- Understorey height<br>- Canopy cover (%)<br>- Intensity of human impact

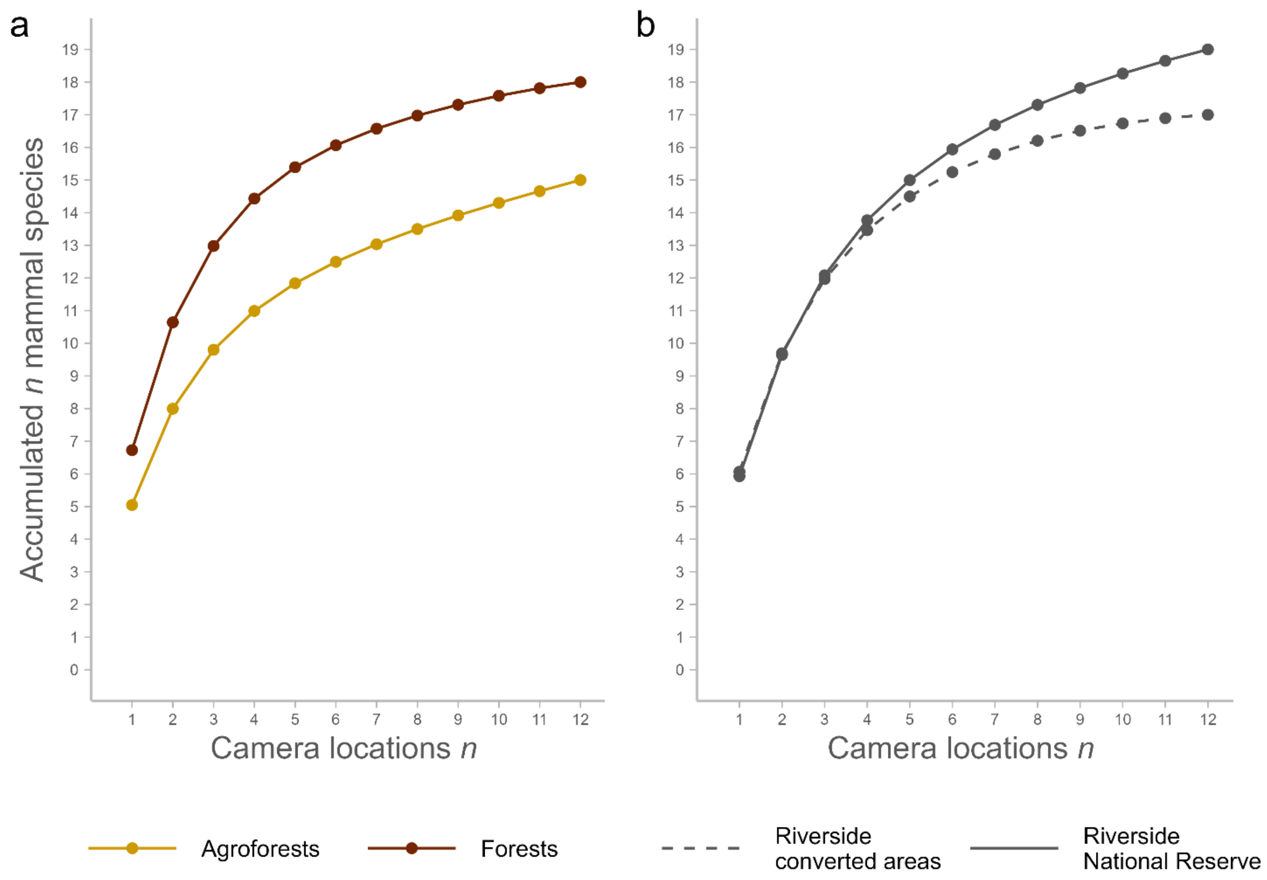

Figure S9. Species accumulation curves. (a) Species accumulation curves by habitat type: agroforests (*n* = 12) shown in orange, and forests (*n* = 12) in brown. (b) Species accumulation curves by riverside: the northern riverside, adjacent to converted areas (*n* locations = 12), marked with a dashed line, and the southern riverside, adjacent to the core zone of Tambopata National Reserve (*n* locations = 12), marked with a solid line.

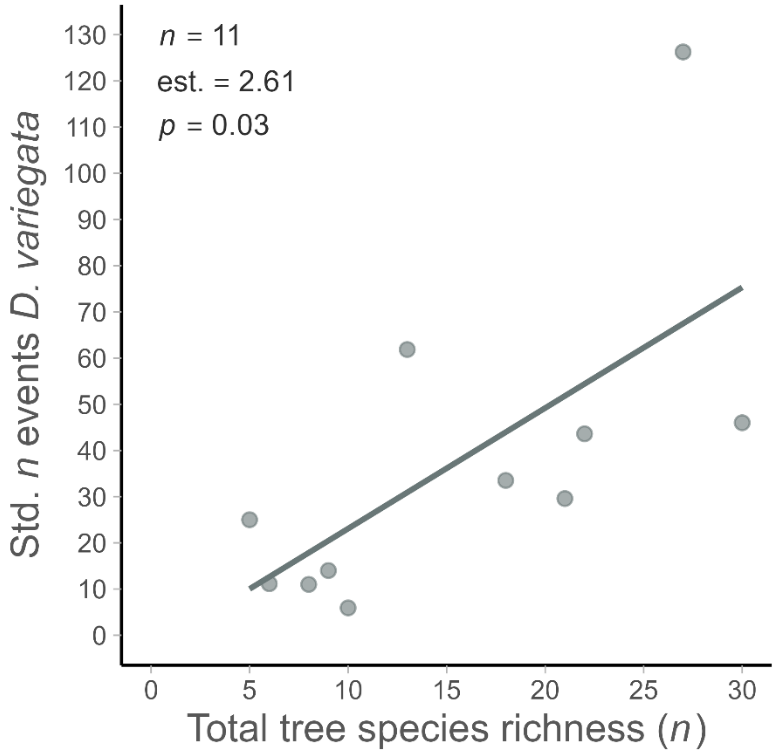

Figure S10. Standardised number of trapping events for brown agouti (*Dasyprocta variegata*) plotted as a function of total tree richness in agroforests. Coefficients in the graph are from linear model: ~ *TreeRich_Total*. Residual standard error = 27.09 on 9 *df*, $R^2_{mult.} = 0.44$, $R^2_{adj.} = 0.38$, F = 7.032 on 1 and 9 *df*, $p = 0.03$.
